# Exploring the Impact of Robotic Hand Rehabilitation on Functional Recovery in Parkinson’s Disease: A Randomized Controlled Trial

**DOI:** 10.3390/brainsci15060644

**Published:** 2025-06-15

**Authors:** Loredana Raciti, Desiree Latella, Gianfranco Raciti, Chiara Sorbera, Mirjam Bonanno, Laura Ciatto, Giuseppe Andronaco, Angelo Quartarone, Giuseppe Di Lorenzo, Rocco Salvatore Calabrò

**Affiliations:** 1 Unità Spinale Unipolare, Azienda Ospedaliera per le Emergenze Cannizzaro, 98102 Catania, Italy; loredana.raciti79@gmail.com; 2 IRCCS Centro Neurolesi Bonino-Pulejo, 98124 Messina, Italy; chiara.sorbera@irccsme.it (C.S.); mirjam.bonanno@irccsme.it (M.B.); laura.ciatto@irccsme.it (L.C.); giuseppe.andronaco@irccsme.it (G.A.); angelo.quartarone@irccsme.it (A.Q.); giuseppe.dilorenzo@irccsme.it (G.D.L.); roccos.calabro@irccsme.it (R.S.C.); 3 Physical Medicine and Rehabilitation, Department of Medical and Surgical Sciences, University of Catanzaro “Magna Graecia”, 88100 Catanzaro, Italy; gianfranco.raciti@gmail.com

**Keywords:** Parkinson’s disease, robotic hand rehabilitation, neurodegenerative disorders, cognitive rehabilitation, upper limb recovery

## Abstract

**Background/Objective:** Parkinson’s disease (PD) is characterized by motor and cognitive impairments that significantly affect quality of life. Robotic-assisted therapies, such as the AMADEO^®^ system, have shown potential in rehabilitating upper limb function but are underexplored in PD. This study aimed to assess the effects of Robotic-Assisted Therapy (RAT) compared to Conventional Physical Therapy (CPT) on cognitive, motor, and functional outcomes in PD patients. **Methods:** A single-blind, randomized controlled trial was conducted with PD patients allocated to RAT or CPT. Participants were assessed at baseline (T0) and post-intervention (T1) using measures including MoCA, FAB, UPDRS-III, 9-Hole Peg Test, FMA-UE, FIM, and PDQ-39. Statistical analyses included ANCOVA and regression models. **Results:** RAT led to significant improvements in global cognition (MoCA, *p* < 0.001) and executive functioning (FAB, *p* = 0.0002) compared to CPT. Motor function improved, particularly in wrist and hand control (FMA-UE), whereas changes in fine motor dexterity (9-Hole Peg Test) were less consistent and did not reach robust significance. No significant improvements were observed in broader quality of life domains, depressive symptoms, or memory-related cognitive measures. However, quality of life improved significantly in the stigma subdomain of the PDQ-39 (*p* = 0.0075). Regression analyses showed that baseline motor impairment predicted cognitive outcomes. **Conclusions:** RAT demonstrated superior cognitive and motor benefits in PD patients compared to CPT. These results support the integration of robotic rehabilitation into PD management. Further studies with larger sample sizes and long-term follow-up are needed to validate these findings and assess their sustainability.

## 1. Introduction

Hand bradykinesia is a hallmark symptom of Parkinson’s disease (PD), contributing substantially to functional disability and reduced quality of life. Among the standardized motor assessments, the finger tapping task from the Movement Disorder Society-Unified Parkinson’s Disease Rating Scale (MDS-UPDRS Part III) is widely used to evaluate the speed, amplitude, and rhythmicity of repetitive index finger movements [1]. Impairments in finger tapping performance, characterized by increased variability and reduced amplitude, have been shown to significantly impact the execution of activities of daily living in patients with Parkinson’s disease [2,3]. These deficits highlight the need for targeted rehabilitation strategies specifically addressing fine motor control of the hand and fingers in PD. Repetitive finger movements have been shown to stimulate cortical plasticity, supporting synaptogenesis and functional reorganization [4,5]. Early, intensive and repetitive training thus emerges as a pivotal strategy to enhance motor relearning and limit motor deficits [6].

Emerging evidence suggests that hand motor impairments in PD are not solely attributable to basal ganglia dysfunction, but also reflect deficits in sensorimotor integration, attention, and executive functions [7,8,9,10]. Fine motor tasks such as finger tapping and object manipulation require dynamic interaction between motor and cognitive systems, including planning, visuospatial coordination, and attentional control. Dual-task studies have shown that PD patients experience greater motor interference when performing simultaneous cognitive tasks, underscoring the interplay between motor performance and cognitive demands [11,12,13,14]. Moreover, limitations in hand function contribute not only to physical disability but also to emotional distress, social withdrawal, and reduced self-efficacy, further impacting overall quality of life [15,16]. In this context, task-specific robotic therapies offer structured, goal-directed activities that require sustained attention and cognitive engagement. By integrating motor and cognitive training, these interventions may support not only the recovery of motor functions but also improvements in mood, motivation, and daily functioning. This highlights the importance of investigating rehabilitation strategies that target both motor and non-motor symptoms.

In the last decades, robotic-assisted therapies have gained increasing attention to deliver high-dose, reproducible, and goal-oriented motor training. Several robotic-assisted therapies have been developed in recent years to support upper limb rehabilitation, especially for hand and finger function. Among the most widely used systems are the HandTutor, which provides task-specific training with biofeedback [17]; the Gloreha, a glove-based exoskeleton that allows for passive and active mobilization combined with visual feedback [18,19]; and the MusicGlove, which integrates musical cues and gamification to enhance repetitive grasp movements and patient engagement [20].

Each device offers specific therapeutic benefits: HandTutor emphasizes precise motion control facilitating motor learning; Gloreha supports muscle reactivation and range of motion improvements, especially in post-stroke patients; while MusicGlove has demonstrated increased motivation and hand function recovery through game-based tasks.

Among them, the AMADEO^®^ robotic system allows for passive, assistive, and active mobilization of individual fingers, offering programmable control of range of motion, speed, and force. Although originally developed for post-stroke rehabilitation, the AMADEO^®^ system has demonstrated excellent reliability in assessing finger strength and spasticity in neurological patients, with strong correlations to established clinical scales [21].

These results reinforce the emerging role of robotics in enhancing both evaluation and rehabilitation processes for upper limb impairments. Indeed, clinical studies and meta-analyses have confirmed the effectiveness of robotic-assisted therapy in improving upper limb function post-neurological injury and promoting neuroplasticity, which is critical for motor recovery [22,23,24]. Despite significant progress in robotic rehabilitation for gait and posture in PD, hand-specific interventions remain underexplored, likely due to the historical lack of fully adaptive, assist-as-needed hand therapies in conventional rehabilitation. In a previous randomized controlled trial, we investigated the effects of a gravity-supporting exoskeleton (Armeo^®^Spring) in patients with Parkinson’s disease compared to conventional physical therapy. The experimental group received 45-min robot-assisted training sessions, 6 days a week for 8 weeks. Results showed significant improvements in hand dexterity (Nine-Hole Peg Test), motor impairment (UPDRS III), and upper limb function (FMA-UE, MI-UE) compared to the control group, suggesting that intensive, repetitive, and task-oriented robotic therapy may effectively enhance upper extremity motor recovery in PD [25].

To our knowledge, no clinical trials have systematically investigated the use of a hand-robotic system in the rehabilitation of patients with PD. Therefore, the aim of this study was to evaluate the efficacy of the AMADEO^®^ robotic device (**Tyromotion GmbH** Bahnhofgürtel 598020 GrazAustria) in enhancing hand and finger function in PD patients.

In addition to motor outcomes, this study comprehensively assessed the impact of the intervention on cognitive functioning, including global cognition, executive functions, working memory, attention, cognitive flexibility, and visuospatial abilities, through an extensive neuropsychological evaluation.

## 2. Materials and Methods

### 2.1. Study Design and Population

We conducted a parallel-group, single blinded, randomized controlled trial involving patients with PD who were managed at the Movement Disorders Clinic of the IRCCS Centro Neurolesi Bonino-Pulejo (Messina, Italy) between July 2019 and December 2023.

Although a double-blind design would help minimize placebo effects and reduce this important source of bias, it is not feasible in rehabilitation settings. In fact, patients randomized to the experimental group are fully aware of receiving robotic-assisted therapy (RAT), making participant blinding impossible. Therefore, only the outcome assessor can be blinded to the treatment allocation.

### 2.2. Inclusion and Exclusion Criteria

Patients were eligible if they: (i) had a confirmed diagnosis of idiopathic PD according to the UK Brain Bank criteria [26]; (ii) had a Hoehn and Yahr stage between 1 and 3 during the “on” phase [27]; (iii) had a Mini-Mental State Examination (MMSE) score of at least 24 [28]; and (iv) were able and willing to provide written informed consent and comply with study procedures.

Exclusion criteria included: diagnosis or suspicion of atypical or secondary Parkinsonism; moderate-to-severe cognitive impairment interfering with task comprehension; severe dyskinesia or marked “on–off” motor fluctuations; prior stereotactic brain surgery for PD; recent changes (within three months prior to baseline) in dopaminergic therapy (measured as levodopa equivalent daily dose—LEDD); and any medical and/or neurological limiting participation in rehabilitation program, such as severe osteoarthritis or peripheral neuropathies.

### 2.3. Ethical Considerations

All participants were informed about the study procedures and provided written informed consent before enrollment. The study was conducted in accordance with national regulations and the principles of Good Clinical Practice (GCP) as well as the Declaration of Helsinki. No external funding or reimbursements were involved.

The study protocol (No. U0074917/11110) received ethical approval from the Ethics Committee of the IRCCS Centro Neurolesi Bonino-Pulejo (Messina, Italy) and was registered at ClinicalTrials.gov (NCT04045080).

### 2.4. Randomization

**Figure 1:**
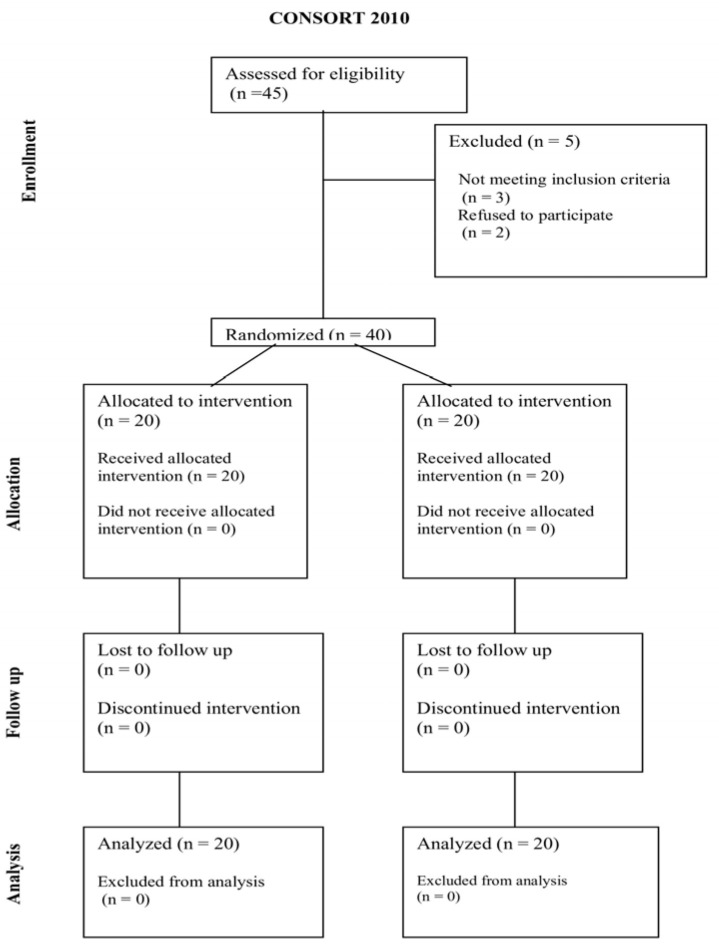
CONSORT 2010 flow diagram.

After the screening phase, patients meeting all inclusion criteria were randomly allocated to either the Robotic-Assisted Therapy (RAT) or the Conventional Physical Therapy (CPT) group. A computerized stratified randomization procedure was employed to ensure balanced group allocation. Stratification was based on the degree of motor impairment, as determined by the Unified Parkinson’s Disease Rating Scale (UPDRS) score [29], to achieve comparable baseline physical capacity across groups. Randomization was performed at a 1:1 ratio using permuted blocks of four to maintain an even distribution of participants between groups. Outcome assessors remained blinded to group assignments throughout the study to minimize assessment bias (see Figure 1).

Figure 1 depicts flow of participants through enrollment, randomization, intervention, and analysis phases, according to CONSORT 2010 guidelines.

### 2.5. Robotic-Assisted Therapy

**Figure 2:**
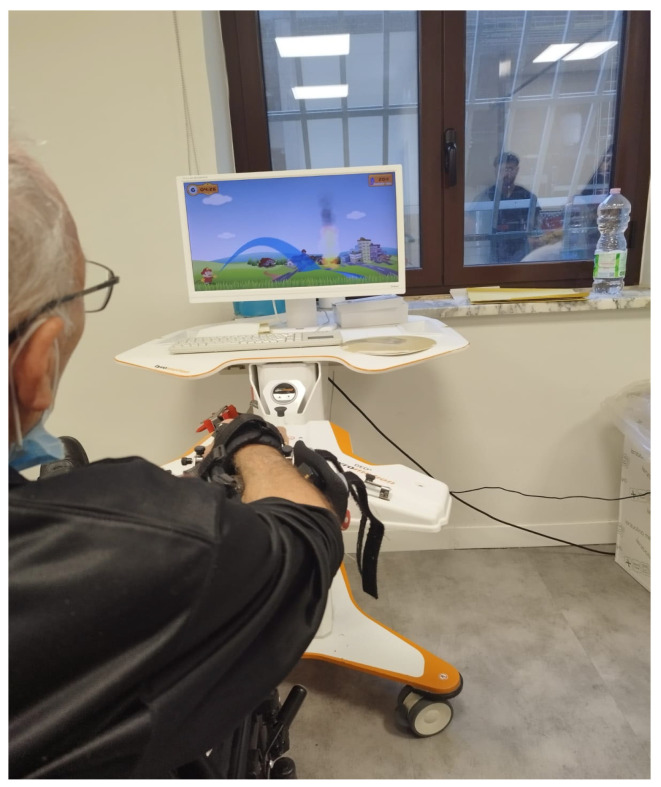
PD patient undergoing robotic-assisted hand therapy using the AMADEO^®^ system.

The AMADEO^®^ system (see Figure 2), developed by Tyromotion GmbH (Graz, Austria), is a state-of-the-art robotic device designed specifically for the rehabilitation of hand and finger function. It is widely used in neurological and orthopedic rehabilitation and is particularly effective for conditions that impair manual dexterity, such as stroke, traumatic brain injury, multiple sclerosis, and Parkinson’s disease.

The key features of the AMADEO^®^ system include:

- Ergonomic Hand Positioning: The patient’s hand is positioned naturally, and each finger is attached individually to a robotic actuator for precise and targeted movement.
- Active and Passive Therapy Modes: The device offers both passive (robot-driven) and active (patient-initiated) modes, supporting rehabilitation across various stages of motor recovery.
- Interactive Feedback and Virtual Reality Integration: AMADEO^®^ incorporates sensory feedback and virtual-reality-based training games to increase patient engagement and motivation. The device’s sensitivity and assistance levels were automatically calibrated before each session based on individual motor performance (finger strength and range of motion), independently of age.
- Real-Time Performance Monitoring: The system collects and displays real-time data on grip strength, range of motion, movement coordination, and therapy efficiency, enabling objective progress tracking and personalized therapy adjustments.

The AMADEO’s sensitivity and assistance levels were customized based on each patient’s condition, providing real-time feedback on key movement parameters such as resistance, strength, range of motion, and coordination. Through its 2D virtual reality interface projected within a 3D space [30], the system promotes movement reinforcement and motor function enhancement.

All training sessions were supervised by a physiotherapist specialized in robotic-assisted rehabilitation. The AMADEO^®^ device was individually calibrated for hand size and suspension angle. Training exercises were personalized, with progressive difficulty adjustments over time. Each therapy session lasted 45 min per arm, 6 d per week, for a total of 8 wk.

### 2.6. Conventional Physical Therapy

Participants assigned to the CPT group underwent conventional rehabilitation sessions of the same duration and frequency as the RAT group. Their therapy included passive and active-assisted mobilization of the upper limbs, neuromuscular facilitation techniques, proprioceptive exercises, and interventions aimed at reducing joint and muscle stiffness. Additionally, patients engaged in active reaching and object-grasping tasks to enhance functional arm use. The intervention was matched in duration and frequency to the robotic-assisted therapy group (See Supplementary Materials).

### 2.7. Outcome Measures

Participants underwent a comprehensive evaluation targeting both motor and non-motor domains, including cognitive, emotional, and quality of life aspects. All assessments were performed at baseline (T0) and after the intervention (T1).

#### 2.7.1. Motor Outcome Measures

Motor function was assessed using the following standardized tools.

#### 2.7.2. Primary Outcome

The 9-Hole Peg Test (9HPT) was selected as the primary outcome measure to assess hand dexterity. Patients were instructed to transfer nine pegs from a container into designated holes and return them as quickly as possible. The primary endpoint was the completion time (in seconds) [31].

#### 2.7.3. Secondary Motor Outcomes

The Unified Parkinson’s Disease Rating Scale (UPDRS-III) [, is a gold standard for assessing motor symptoms in PD [32].

Fugl-Meyer Assessment for Upper Extremity (FMA-UE) [] evaluates selective motor control across shoulder, elbow, wrist, hand, and coordination. It evaluates selective voluntary motor control in the upper limb. The scale consists of 33 items scored on a 3-point ordinal scale (0–2), yielding a maximum score of 66 points. Subdomains assess movements of the upper arm (36 points), wrist (10 points), hand (14 points), and coordination/speed (6 points) [33].

All motor assessments and therapeutic interventions were conducted bilaterally, considering both the more and less affected side. Moreover, cognitive assessment was performed to evaluate improvement on executive, memory, or mood issues.

#### 2.7.4. Neuropsychological Outcome Measures

Non-motor outcomes included cognitive performance, mood, and quality of life, assessed through:

The Montreal Cognitive Assessment (MoCA) is a screening tool for global cognitive function, evaluating memory, attention, language, executive functions, and visuospatial skills [34].

The Frontal Assessment Battery (FAB) is a brief bedside tool assessing executive functions, including conceptualization, mental flexibility, and inhibitory control [35].

The Wisconsin Card Sorting Test (WCST) is a measure of cognitive flexibility, set-shifting, and problem-solving abilities [36].

Verbal Fluency Tests assess lexical retrieval and executive functions by requiring the generation of words within specific categories and starting with a given letter [37].

The Rey–Osterrieth Complex Figure Test measures visuospatial constructional ability and visual memory through the reproduction of a complex geometric figure [38].

Digit Span (Forward and Backward) assesses short-term memory and working memory by recalling sequences of numbers in the presented or reverse order [39].

The Trail Making Test (TMT) Parts A and B evaluates processing speed (Part A) and cognitive flexibility/set-shifting (Part B) by connecting sequences of numbers or alternating numbers and letters [40].

#### 2.7.5. Behavioral Outcome Measures 

The Hamilton Depression Rating Scale (HAM-D) was used to assess the severity of depressive symptoms, with validated cutoff scores for identifying depressive symptoms and major depressive disorder. It has been shown to have good sensitivity and specificity. Cutoff scores of 9/10 and 11/12 to screen for dPD, and 15/16 and 13/14 to diagnose major depressive disorder (although diagnosis using a scale alone is not recommended) have been suggested [41].

The Parkinson’s Disease Questionnaire-39 (PDQ-39), a PD-specific health status questionnaire, assessed the impact of PD across eight domains. Scores were normalized to a 0–100 scale, with higher scores indicating worse health status [42].

#### 2.7.6. Statistical Analysis

Statistical analysis was conducted using IBM SPSS Statistics version 29.0.2.0 (IBM Corp., Armonk, NY, USA) and STATA 10 software packages (StataCorp. 2011. Stata Statistical Software: Release 10. College Station, TX, USA: StataCorp LP). Descriptive statistics (mean, standard deviation) were used to compare demographic variables (age, sex, education) between the experimental and control groups. Independent samples *t*-tests and Chi-square tests were used to assess group homogeneity. Paired samples *t*-tests were employed to compare pre- and post-intervention scores (T0 vs. T1) within each group. Between-group comparisons of change scores (T1–T0) were performed using independent samples *t*-tests. To control baseline differences, ANCOVA (analysis of covariance) models were computed, with post-intervention scores as the dependent variable, group as the fixed factor, and baseline scores as covariates. Multiple linear regression models were used to explore the predictive value of clinical and demographic variables (age, sex, education, H&Y stage, UPDRS III T0) on outcomes at T1. To complement the histogram of age distribution, a Kernel Density Estimation (KDE) curve was added. KDE is a non-parametric method that estimates the probability density function of a continuous variable, providing a smoothed approximation of the data distribution without assuming a specific parametric form.

## 3. Results

### 3.1. Baseline Characteristics

**Figure 3:**
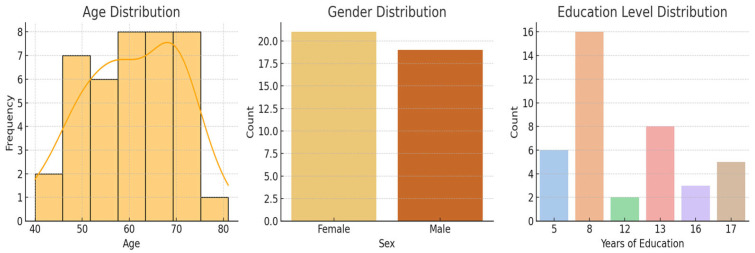
Combined demographic distributions. Legend: The left panel shows the histogram of participants’ age with a KDE curve; The Y-axis in the age distribution plot indicates the absolute frequency, i.e., the number of individuals within each age bin; the middle panel displays the gender distribution; the right panel presents the count of participants by years of education.

The analysis of demographic variables revealed significant differences between groups. Participants in the experimental group were older (64.8 vs. 56.8 years; *p* = 0.01) and had completed fewer years of education (8.8 vs. 12.2; *p* = 0.007) compared to the control group (Table 1, Figure 3). No significant baseline differences were observed for MoCA, UPDRS III, or 9HPT scores (Table 2).

### 3.2. Post-Intervention Between-Group Comparisons

**Figure 4:**
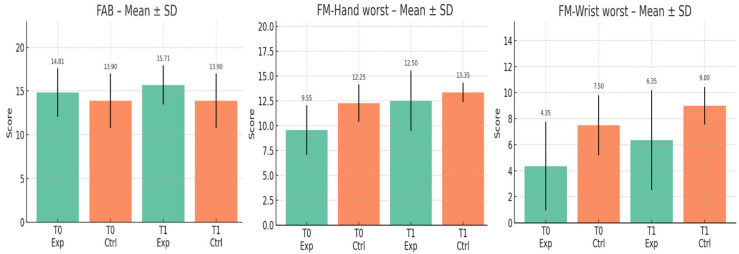
Grouped bar plots of outcome measures showing significant differences between groups or timepoints (Mean ± SD). Legend: mean ± standard deviation (SD) for each test at T0 and T1, comparing the experimental and control groups. FAB T0 experimental 14.81 ± 2.79 vs. control groups 13.90 ± 3.11, *p*-value = 0.3; FAB T1 experimental 15.71 ± 2.24 vs. control groups 13.90 ± 3.11, *p*-value: 0.04; FM hand affected side T0: experimental 9.55 ± 2.50 vs. control group 12.25 ± 1.89, *p*-value: 0.0005; FM hand affected side T1: experimental 12.50 ± 3.05 vs. control group 13.35 ± 0.99, *p*-value: 0.2; FM wrist affected side T0: experimental 4.35 ± 3.41 vs. control group 7.50 ± 2.31, *p*-value: 0.0016; FM wrist affected side T1: experimental 6.35 ± 3.84 vs. control group 9.00 ± 1.45, *p*-value: 0.0081.

At T1, most outcomes did not differ significantly between groups. MoCA, UPDRS III, and 9HPT scores remained comparable (Table 2). It is important to note that Table 2 presents inter-group comparisons at each time point (T0 and T1), whereas Table 3 reports intra-group changes from pre- to post-intervention within the experimental group. Therefore, the presence or absence of significance in Table 2 does not reflect the effect of the intervention on individual group performance. However, the FMA-UE wrist subscore (affected side) was significantly higher in the experimental group at both baseline (*p* = 0.0016) and T1 (*p* = 0.008) (Figure 4).

### 3.3. Within-Group Improvements in the Experimental Group

Significant post-intervention improvements were observed in the experimental group for motor function (UPDRS III; *p* = 0.000), FMA-UE hand and wrist (*p* = 0.0001, *p* = 0.0006), coordination (*p* = 0.0002), and 9HPT (*p* = 0.03). Cognitive scores also improved, with MoCA (*p* = 0.000) and FAB (*p* = 0.0027) both increasing significantly. Additionally, the stigma subscale of PDQ-39 showed a reduction (*p* = 0.0075), while other domains of quality of life and cognitive function showed no significant changes. A near-significant improvement was noted in phonemic fluency (*p* = 0.05).

### 3.4. Exploratory Regression Analysis

**Figure 5:**
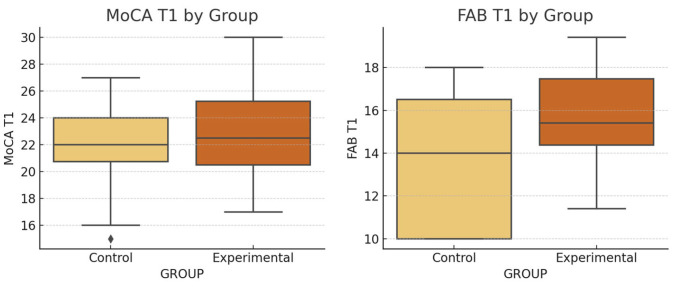
Group comparison of post-intervention (T1) scores using ANCOVA, adjusted for baseline values. Legend: Boxplots of MoCA and FAB scores at T1 for the control and experimental groups. The experimental group shows higher median scores in both tests. Differences are consistent with ANCOVA results (*p* = 0.0002 for both tests).

**Figure 6:**
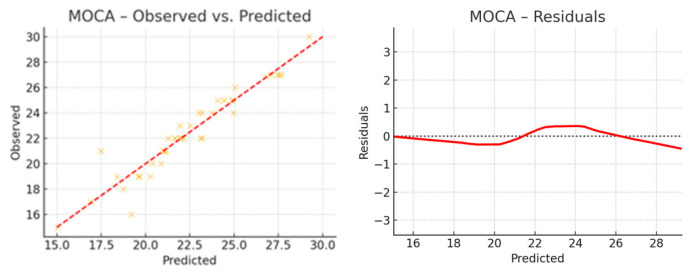
Regression analysis of MoCA scores: observed vs. predicted values and corresponding residual plot. Legend: This scatterplot shows how closely predicted values from the regression model align with the observed post-intervention scores. The left panel shows the regression of MoCA post-intervention scores: observed vs. predicted values, with the identity line (y = x) representing perfect prediction. The right panel displays standardized residuals to evaluate the goodness of fit and potential outliers. A random distribution around zero supports model validity. MoCA—observed vs. predicted.

**Figure 7:**
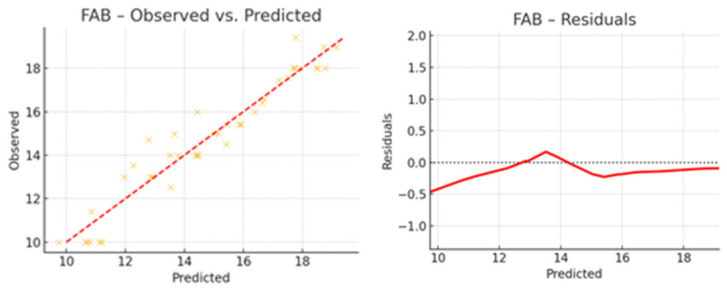
Regression analysis of FAB scores: observed vs. predicted values and residual distribution. Legend: This scatterplot shows how closely predicted values from the regression model align with the observed post-intervention scores. The left panel presents the regression plot for FAB post-intervention scores: observed vs. predicted values with reference line (y = x). The right panel illustrates the standardized residuals, used to assess model adequacy and residual variance. A random distribution around zero supports model validity. FAB—observed vs. predicted.

**Figure 8:**
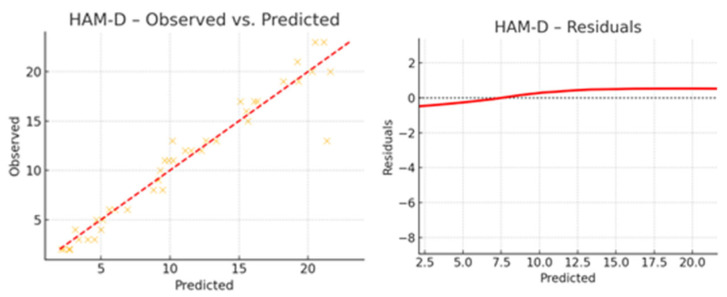
Regression analysis of HAM-D scores: observed vs. predicted values and residual distribution. Legend: This scatterplot shows how closely predicted values from the regression model align with the observed post-intervention scores. The left panel depicts the regression of HAM-D post-intervention scores: observed vs. predicted values, compared to the identity line. The right panel shows the residual distribution, highlighting potential deviations and variance patterns A random distribution around zero supports model validity. HAM-D—observed vs. predicted.

**Figure 9:**
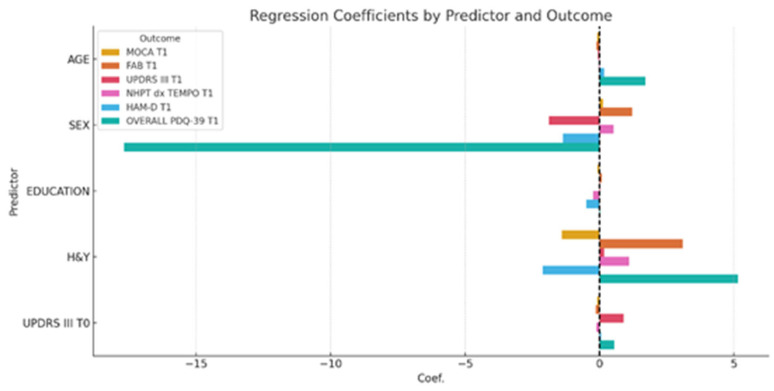
Standardized regression coefficients of significant predictors for post-intervention outcomes. Legend: Each bar represents the influence (coefficient) of a predictor on an outcome. The color identifies the outcome (e.g., MOCA T1, FAB T1…). The horizontal axis shows the strength and direction of the effect. Bars to the right: positive influence; Bars to the left: negative influence. The dashed vertical line at 0 separates positive from negative effects. This graph visually summarizes how each variable contributes (positively or negatively) to the different outcomes, allowing comparison across domains.

The regression analysis aimed to identify which clinical factors predict the outcomes observed at T1 (post-intervention). The variables included as predictors were age, sex, education, H&Y, UPDRS III at T0 and T1. The dependent variables were MoCA, FAB, UPDRS III, 9HPT (dexterity), HAM-D, and the PDQ-39 total score at T1 (Table 4). The regression analysis revealed that higher education was positively associated with better performance on cognitive measures such as MoCA and FAB at T1. Conversely, older age and greater baseline motor severity (as measured by UPDRS III) were negatively associated with several cognitive, motor, and quality-of-life measures. H&Y stage showed a modest negative impact across motor and quality-of-life measures. The influence of sex was generally less pronounced, although in some outcomes it displayed minor predictive weight. An initial, general regression model across all outcomes showed moderate explained variance (R^2^ ≈ 0.34), providing a global view of predictor–outcome relationships. Also, regression analysis revealed a moderate variance (R^2^ ≈ 34%), with no major confounding factors identified. However, when building outcome-specific models (MoCA, FAB, HAM-D), the explanatory power increased considerably, with R^2^ values ranging from 0.91 to 0.93, as shown in Table 4 and Figure 5. These results support the conclusion that the rehabilitation program exerted a real and measurable positive effect on global cognitive function. In these models, greater baseline motor severity (UPDRS III T0) and higher H&Y stage were associated with worse dexterity (9HPT) and poorer quality of life (PDQ-39). The regression results revealed strong models with high explanatory power for all three outcomes. For MOCA T1, the model explained 91.1% of the variance (R^2^ = 0.911). Significant predictors were baseline MOCA, FAB, and HAM-D scores, indicating that better baseline cognitive and mood performance predicted higher post-treatment global cognition. For FAB T1, the model explained 92.7% of the variance (R^2^ = 0.927), with baseline FAB as the only significant predictor, suggesting strong test–retest consistency and specificity in executive function. For HAM-D T1, the model explained 92.9% of the variance (R^2^ = 0.929), with baseline HAM-D as the sole significant predictor, reflecting stability in depressive symptomatology over time. These findings indicate that baseline characteristics and demographic characteristics may influence post-intervention performance and merit further investigation. Regression results are summarized in Table 4 and visually represented in Figure 6, Figure 7 and Figure 8, which show the observed versus predicted values and residual plots for each outcome. The tight clustering along the identity line and low residual variance confirms the robustness of the regression models. Figure 9 illustrates the standardized regression coefficients for each predictor variable across the selected T1 outcomes. Bars extending to the right of the vertical zero line represent positive associations, while those extending to the left indicate negative associations.

Positive standardized coefficients indicate that higher values of the predictor are associated with better outcomes (e.g., higher cognitive scores), whereas negative coefficients suggest that higher predictor values are associated with poorer outcomes (e.g., increased disability or reduced quality of life).

This bidirectional representation provides a clear overview of how each baseline variable contributed to cognitive, motor, and emotional outcomes following the intervention.

## 4. Discussion

This randomized controlled trial investigated the impact of RAT compared to CPT on motor, cognitive, and functional in patients with PD.

At baseline, the two groups were largely comparable, except for minor differences in age and education, which were statistically adjusted in the analyses. After intervention, the RAT group demonstrated significant improvements in global cognition, as measured by the MoCA, and in executive functioning, as per FAB. These improvements were confirmed by ANCOVA analyses, highlighting that the effects were independent of baseline disparities.

The observed improvement in global cognitive functioning (MoCA: +1.8 points, *p* < 0.001) in the experimental group suggests that robotic-assisted therapy may effectively support frontal cognitive engagement during motor training. This is particularly relevant in Parkinson’s disease, where executive dysfunction is a common non-motor symptom and contributes significantly to loss of independence. Executive functioning, as assessed by FAB, also improved significantly (+0.9 points, *p* = 0.0027), reinforcing the hypothesis that interactive, feedback-rich rehabilitation can stimulate cognitive flexibility and inhibitory control—functions often linked to prefrontal cortex integrity. This supports the clinical rationale for integrating cognitive–motor interventions in multidomain rehabilitation programs.

The significant improvement observed in FAB scores (*p* = 0.0002) in our study supports the concept that targeted interactive rehabilitation strategies, such as robotic therapy with VR, may offer superior cognitive benefits compared to traditional physical therapy alone [43,44,45].

Indeed, it is believed that robotic intervention may have specifically stimulated frontal–executive circuits involved in motor planning, inhibitory control, and cognitive flexibility [46,47].

The repetitive, goal-directed, and attentionally demanding nature of the robotic exercises may have contributed to reinforcing top-down executive control, which is often compromised in PD. This aligns with emerging evidence suggesting that combining cognitive and motor tasks can promote cross-domain neuroplasticity, particularly within frontal–striatal networks. These findings are consistent with prior studies suggesting that cognitive training combined with physical therapy may enhance executive function in PD patients. For instance, Johansson et al. [48] demonstrated that dual-task training improves cognitive flexibility and processing speed in PD. Similarly, Strouwen et al. [49] showed that cognitive–motor interventions have synergistic effects on executive control.

Regarding motor function, the experimental group showed significant improvements in selective upper limb movement control (Fugl–Meyer scores). Improvements in upper limb motor performance were evident in the experimental group, particularly in hand and wrist function (FMA-UE Hand: +2.95 points, *p* = 0.0001; Wrist: +2.0 points, *p* = 0.0006). These gains suggest that distal-focused robotic training can contribute meaningfully to motor recovery, even in chronic neurodegenerative conditions [50,51]. Although improvements in wrist and hand control were observed, the results on fine motor dexterity (9HPT) were less consistent. This may be due to baseline differences between groups and the limited sample size, which might have reduced the sensitivity of this specific measure. This unexpected trend might be explained by subjective perceptions, external environmental factors, or non-specific effects unrelated to the treatment itself. Therefore, while the difference is statistically significant, caution is warranted in interpreting it as a direct effect of the intervention. Importantly, these motor improvements are consistent with those observed in our previous work using the Armeo^®^Spring [25], where gravity-supported, semi-autonomous training led to significant gains in dexterity, upper limb function, and overall disability burden in PD patients. In that study, the intensive, repetitive, and task-oriented training provided by the exoskeleton was hypothesized to trigger profound cortical and subcortical neuroplastic changes, involving both primary motor areas and compensatory cerebellar pathways. Similarly, earlier studies conducted with the Amadeo^®^ robotic device [52], specifically targeting fine finger movements, revealed that task-specific fine motor training can induce selective cortical reorganization, particularly within the sensorimotor hand representation areas.

These results reinforce the hypothesis that robotic rehabilitation, whether focused on proximal (Armeo^®^Spring) or distal (Amadeo^®^) segments, is capable of engaging neuroplastic mechanisms at multiple hierarchical levels of the motor system. Furthermore, the cognitive gains observed in the present study suggest that robotic training may also have a positive impact on frontal–executive networks, supporting the increasingly recognized link between motor recovery and cognitive improvement in PD [53]. A significant reduction in PDQ-39 stigma scores (*p* = 0.0075) suggests that robotic training may positively impact patients’ self-perception and reduce feelings of social embarrassment. This psychosocial benefit, while secondary, is clinically meaningful and aligns with the growing emphasis on patient-centered outcomes in neurorehabilitation. This finding may reflect a reduction in patients’ perceived social isolation or self-stigmatization, potentially mediated by improved self-efficacy and engagement in structured, technology-mediated rehabilitation. Robotic therapy may offer a motivational and socially validating experience, counteracting feelings of incompetence or embarrassment commonly reported by individuals with PD. Such psychosocial benefits, although secondary, are clinically meaningful and support the integration of patient-centered, engaging interventions in rehabilitation programs. These findings mirror those of studies like that of Schenkman et al. [54], which emphasized the importance of integrated rehabilitation in improving PD patients’ perceived social integration and autonomy. Regression analyses indicated that baseline motor impairment (UPDRS III) was predictive of executive function outcomes, suggesting a strong interplay between motor and cognitive domains in PD, as previously theorized by Kehagia et al. [55]

While significant improvements were observed in motor and executive domains, several secondary outcomes such as depressive symptoms, general quality of life, and verbal memory did not show significant changes. This may reflect the domain-specific action of the AMADEO^®^ device, which primarily targets fine motor function and sensorimotor integration. These results are consistent with previous findings suggesting that robot-assisted therapy produces localized improvements in motor and executive functioning, whereas broader cognitive or emotional outcomes often require multi-component interventions or longer durations [5,50,51]. Upper limb dexterity and overall quality of life demonstrated less consistent or less reliable evidence of a direct treatment effect, highlighting the need for cautious interpretation in these areas. From a psychometric standpoint, internal consistency across outcome measures was moderate (Cronbach’s α = 0.615). Principal component analysis (PCA) showed that the outcome measures reflect different domains—motor, cognitive, and emotional—rather than a single dimension. This supports the need for multidomain rehabilitation strategies in Parkinson’s disease. To summarize, the most robust and reliable evidence of rehabilitation-induced improvement was observed in the domains of global cognitive functioning and executive functioning. Specifically, patients in the experimental group exhibited improvements in MoCA scores and significant improvements in FAB performance, particularly among individuals with milder baseline motor impairment. These findings are consistently supported by multiple analytical approaches, including pre/post comparisons, ANCOVA models controlling for baseline differences, and regression analyses accounting for relevant clinical covariates. In contrast, other domains, such as upper limb dexterity and overall quality of life, demonstrated less consistent or less reliable evidence of a direct treatment effect, highlighting the need for cautious interpretation in these areas.

### Strengths and Limitations

A major strength of this study is the rigorous design, including stratified randomization and blinded outcome assessment. However, limitations must be acknowledged. The relatively small sample size may have limited statistical power, particularly for secondary outcomes. However, demographic factors (age, education, sex) showed limited influence overall. Minor baseline differences and absence of long-term follow-up restrict generalizability. The subjective nature of some measures (e.g., PDQ-39) may have introduced reporting bias.

## 5. Conclusions

In conclusion, this study provides preliminary evidence that RAT not only improves selective motor skills but also significantly enhances cognitive functions, especially executive abilities, in individuals with PD. These results support the growing body of literature suggesting that intensive, technology-assisted rehabilitation can offer superior outcomes compared to conventional approaches. Future larger-scale, longitudinal studies are warranted to confirm these findings and explore the sustainability of cognitive and motor gains over time.

## Figures and Tables

**Table 1:** Descriptive comparison between groups.

Variable	Experimental (Mean ± SD, n = 20)	Control (Mean ± SD, n = 20)	*p*-Value
Age	64.8 ± 9.04	56.8 ± 9.96	0.01
Education (years)	8.80 ± 3.58	12.2 ± 3.86	0.007
Sex (M/F)	9 F (11 M)	12 F (8 M)	0.5266

**Table 2:** Inter-group comparison at T0 and T1.

Test	Experimental T0 (Mean ± SD, n = 20)	Control T0 (Mean ± SD, n = 20)	*p*-Value T0	Experimental T1 (Mean ± SD, n = 20)	Control T1 (Mean ± SD, n = 20)	*p*-Value T1
MoCA	21.09 ± 4.29	21.85 ± 3.03	0.5	22.89 ± 3.56	22.10 ± 3.11	0.5
UPDRS III	33.95 ± 8.06	34.05 ± 12.80	0.9	27.95 ± 6.52	31.65 ± 11.94	0.2
FAB	14.81 ± 2.79	13.90 ± 3.11	0.3	15.71 ± 2.24	13.90 ± 3.11	0.04
HAM-D	15.10 ± 7.28	7.85 ± 5.17	0.0009	13.95 ± 6.28	7.85 ± 5.17	0.002
PDQ-39	63.50 ± 27.7	66.2 ± 29.7	0.8	62.20 ± 28.28	58.90 ± 29.2	0.7
9HPT affected side	32.45 ± 18.1	35.99 ± 5.94	0.6	30.1 ± 18.98	31.89 ± 4.12	0.7
FMA-UE hand affected side	9.55 ± 2.50	12.25 ± 1.89	0.0005	12.50 ± 3.05	13.35 ± 0.99	0.2
FMA-UE wrist affected side	4.35 ± 3.41	7.50 ± 2.31	0.0016	6.35 ± 3.84	9.00 ± 1.45	0.0081

Legend: MoCA = Montreal Cognitive Assessment; UPDRS III: Unified Parkinson’s Disease Rating Scale third subscale; FAB = Frontal Assessment Battery; HAM-D = Hamilton Depression Rating Scale; PDQ-39 = Parkinson’s Disease Questionnaire-39; 9HPT = 9-Hole Peg Test; FMA-UE = Fugl–Meyer Assessment.

**Table 3:** Experimental group—T0 vs. T1 comparison (n = 20).

Test	Mean T0 ± SD	Mean T1 ± SD	*p*-Value (T0 vs. T1)
Motor assessment			
UPDRS III	33.95 ± 8.06	27.95 ± 6.52	0.0
FMA-UE Hand affected side	9.55 ± 2.50	12.50 ± 3.05	0.0001
FMA-UE Wrist affected side	4.35 ± 3.41	6.35 ± 3.84	0.0006
FMA-UE coordination	3.70 ± 1.59	4.70 ± 1.53	0.0002
9HPT affected side	32.45 ± 18.06	30.05 ± 18.98	0.03
Cognitive assessment			
MOCA	21.09 ± 4.29	22.89 ± 3.56	0.0
FAB	14.81 ± 2.79	15.71 ± 2.24	0.0027
HAM-D	15.10 ± 7.28	13.95 ± 6.28	0.1
PDQ-39	63.50 ± 27.68	62.20 ± 28.28	0.5
PDQ-39 ADL	43.3 ± 29.6	43.5 ± 27.2	0.9
PDQ-39 stigma	25.6 ± 23.9	20.6 ± 25.7	0.0075
PDQ-39 SS	15.4 ± 29.6	16.2 ± 29.9	0.7
PDQ-39 CI	39.1 ± 15	39.1 ± 15.9	1.0
PDQ-39 com	40 ± 20.7	39.2 ± 21.8	0.8
DIGIT SPAN	6.92 ± 1.87	7.22 ± 1.60	0.4
ROCF copy	18.74 ± 9.86	19.94 ± 9.22	0.5
ROCF IR	10.02 ± 9.24	8.80 ± 6.38	0.4
ROCF DR	11.38 ± 12.24	9.96 ± 6.80	0.6
Phonemic fluency	29.14 ± 12.25	32.19 ± 13.69	*0.05*
Semantic fluency	35.10 ± 11.22	37.15 ± 11.47	0.09
Attentive matrices	38.30 ± 12.47	41.34 ± 10.41	0.09
TMT A	60.70 ± 37.38	62.30 ± 34.55	0.8
TMT B	81.10 ± 77.42	93.90 ± 66.45	0.2
TMT B-A	47.45 ± 46.87	43.25 ± 47.28	0.6
WCST	32.96 ± 31.59	36.91 ± 35.56	0.3

Legend: FMA-UE = Fugl–Meyer Assessment Upper Extremity; 9HPT = 9-Hole Peg Test; UPDRS III: Unified Parkinson’s Disease Rating Scale third subscale; MoCA = Montreal Cognitive Assessment; FAB = Frontal Assessment Battery; HAM-D = Hamilton Depression Rating Scale; PDQ-39 ADL = Parkinson’s Disease Questionnaire-39, Activities of Daily Living subscale; PDQ-39 Stigma = Parkinson’s Disease Questionnaire-39, Stigma subscale; PDQ-39 SS = Parkinson’s Disease Questionnaire-39, Social Support subscale; PDQ-39 CI = Parkinson’s Disease Questionnaire-39, Cognitive Impairment subscale; PDQ-39 Com = Parkinson’s Disease Questionnaire-39, Communication subscale; ROCF Copy = Rey–Osterrieth Complex Figure Test, Copy phase; ROCF IR = Rey–Osterrieth Complex Figure Test, Immediate Recall phase; ROCF DR = Rey–Osterrieth Complex Figure Test, Delayed Recall phase; TMT A = Trail Making Test Part A; TMT B = Trail Making Test Part B; TMT B-A = Difference between TMT B and TMT A; WCST = Wisconsin Card Sorting Test.

**Table 4:** Regression analyses: Observed vs. predicted and residuals.

Outcome	R-Squared	Significant Predictors
MOC A	0.911	MOCA_T0, FAB_T0, HAM-D_T0
FAB	0.927	FAB_T0
HAM-D	0.929	HAM-D_T0

## Data Availability

The original data presented in the study are openly available in Zenodo Repository at https://zenodo.org/records/15462063 (accessed on 19 May 2025).

## Supplementary Materials

The following supporting information can be downloaded at: https://www.mdpi.com/article/10.3390/brainsci15060644/s1, Table S1: Description of both RAT and CPT rehabilitation protocol and exercises
