# Supplementary material for: Exploring the Impact of Robotic Hand Rehabilitation on Functional Recovery in Parkinson’s Disease: A Randomized Controlled Trial
Source: Brain Sci. 2025 Jun 15;15(6):644. doi: 10.3390/brainsci15060644 (PMC12190367; doi:10.3390/brainsci15060644)
Supplement: Supplementary file 1 [file brainsci-15-00644-s001.zip › brainsci-3673789-supplementary.pdf]

Supplementary Table S1. Description of both RAT and CPT rehabilitation protocol and exercises.

RAT Activity and Games	RAT Exercise	CPT Activity	CPT Exercise	Target movement of RAT and CPT	Exercise Time
**Elbow, wrist and fingers stretching**	Stretching by the therapist of the biceps brachii and brachioradialis, pronator, flexor carpi ulnaris and radialis, palmaris longus, flexor digitorum superficial and deep, flexor pollicis longus and adductor.	Elbow, wrist and fingers stretching	Stretching by the therapist of the biceps brachii and brachioradialis, pronator, flexor carpi ulnaris and radialis, palmaris longus, flexor digitorum superficial and deep, flexor pollicis longus and adductor.	NA	10′
**Elevator Operating**	Elevator in a building, people must be picked up and taken to the correct floor	Jewels	The patient must create necklaces and bracelets, inserting various shapes of pasta into the string.	Flexion and extension of fingers	7′
**Applehunter**	Falling apples must be caught with a basket	Basket	The patient must try to make a basket by throwing tinfoil balls.	Flexion and extension of fingers	7′
**Firefighters**	Flaring flames must be extinguished with a water jet as precisely as possible, achieving and maintaining the required strength and/or motion level.	Maze	The patient must follow the right path with his fingers to solve the maze.	Flexion and extension of fingers	7′
**Cars**	Steering a vehicle in traffic	Cars	The patient moves a toy car among obstacles on a table.	Flexion and extension of fingers	7′

**Ballon**	Maneuvering a balloon through a course and past obstacles	Bowling	The patient positioned in front of a table must throw a ball and hit the pins to make a strike	Flexion and extension of fingers	7′
